# Supplementary material for: WNT signaling in human pluripotent stem cells promotes HDAC2-dependent epigenetic programs and development of retinoic acid-responsive mesoderm
Source: bioRxiv. 2025 Jun 8:2025.06.06.657928. Preprint. [Version 1] doi: 10.1101/2025.06.06.657928 (PMC12258972; doi:10.1101/2025.06.06.657928)
Supplement: 1 [file NIHPP2025.06.06.657928v1-supplement-1.pdf]

bioRxiv preprint doi: https://doi.org/10.1101/2025.06.06.657928; this version posted June 8, 2025. The copyright holder for this preprint (which was not certified by peer review) is the author/funder, who has granted bioRxiv a license to display the preprint in perpetuity. It is made available under aCC-BY 4.0 International license.

Fig. S1

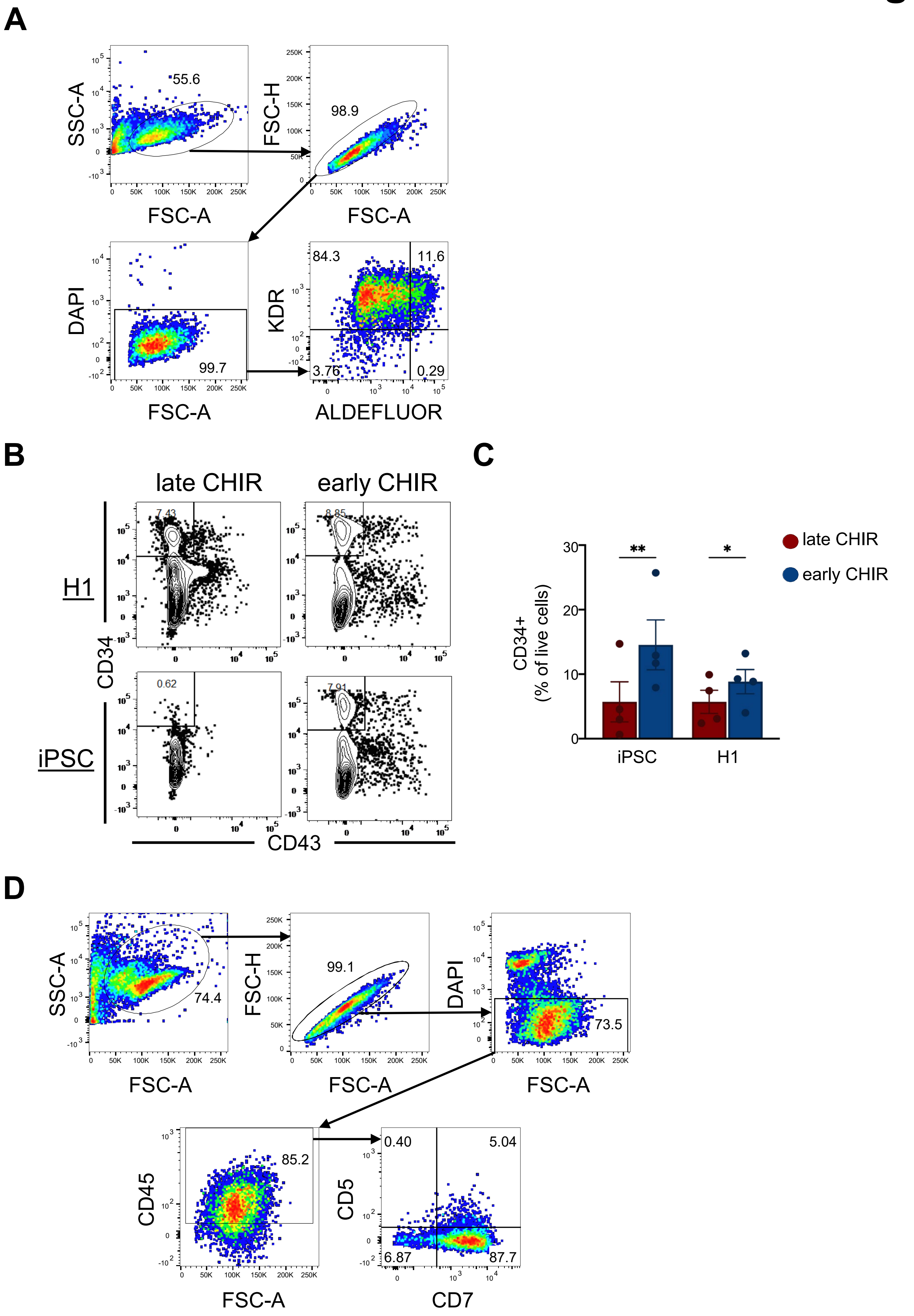

bioRxiv preprint doi: https://doi.org/10.1101/2025.06.06.657928; this version posted June 8, 2025. The copyright holder for this preprint (which was not certified by peer review) is the author/funder, who has granted bioRxiv a license to display the preprint in perpetuity. It is made available under aCC-BY 4.0 International license.

Figure S1 related to Figure 1. Differentiation of mesodermal progenitors from hPSCs and the role of CHIR 99021 in WNT signaling activation. (A) Gating strategy for flow cytometry analysis of KDR and ALDEFLUOR expression in H1- and iPSC-derived progenitors under early or late CHIR conditions. (B) Representative flow cytometry plots showing expression of CD34 and CD43 in H1- and iPSC- derived hematopoietic progenitors on day 16 of EB differentiation, with early or late CHIR treatments. (C) Quantification of the percentage of CD34+ cells in H1- and iPSC-derived cultures. Statistical analysis was performed using 2-way ANOVA with Šídák multiple comparisons test and $^{*}p < 0.05$, $^{**}p < 0.01$. Data are presented as mean ± SEM. (D) Gating strategy for flow cytometry analysis of CD5 and CD7 expression in H1- and iPSC-derived progenitors.

bioRxiv preprint doi: https://doi.org/10.1101/2025.06.06.657928; this version posted June 8, 2025. The copyright holder for this preprint (which was not certified by peer review) is the author/funder, who has granted bioRxiv a license to display the preprint in perpetuity. It is made available under aCC-BY 4.0 International license.

Fig. S2

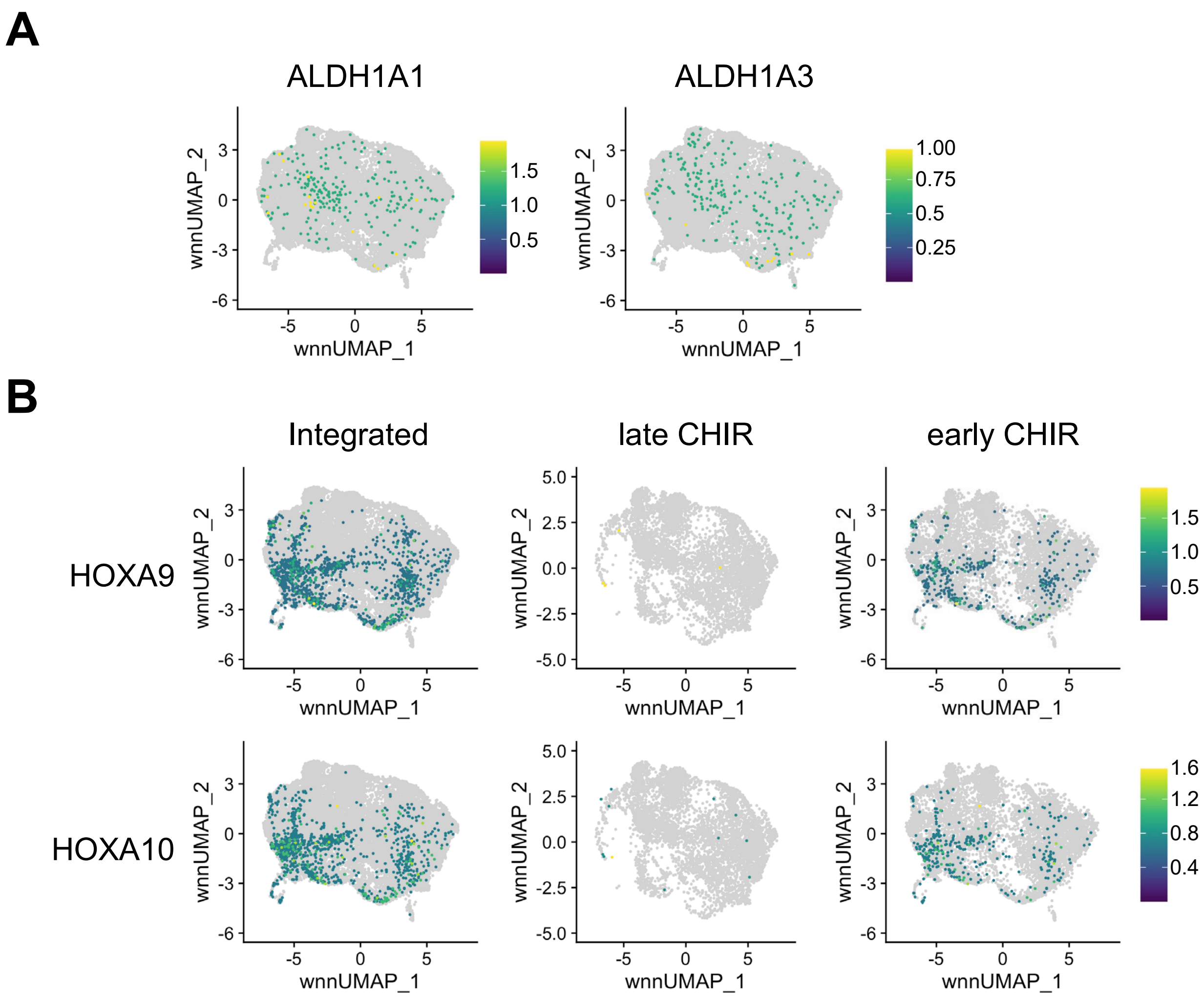

bioRxiv preprint doi: https://doi.org/10.1101/2025.06.06.657928; this version posted June 8, 2025. The copyright holder for this preprint (which was not certified by peer review) is the author/funder, who has granted bioRxiv a license to display the preprint in perpetuity. It is made available under aCC-BY 4.0 International license.

Figure S2 related to Figure 2. WNT signaling induces transcriptional changes associated with ALDH1A2- and HOXA-specific hematopoietic programs. (A) Relative expression of ALDH1A1 and ALDH1A3. (B) UMAP plots showing the relative expression of *HOXA9* and *HOXA10*.

bioRxiv preprint doi: https://doi.org/10.1101/2025.06.06.657928; this version posted June 8, 2025. The copyright holder for this preprint (which was not certified by peer review) is the author/funder, who has granted bioRxiv a license to display the preprint in perpetuity. It is made available under aCC-BY 4.0 International license.

Fig. S3

A

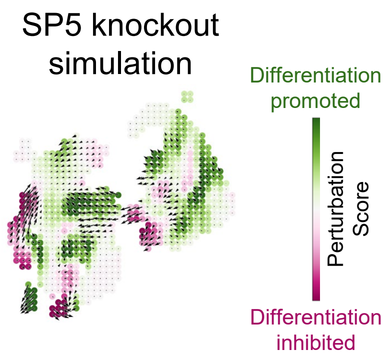

B

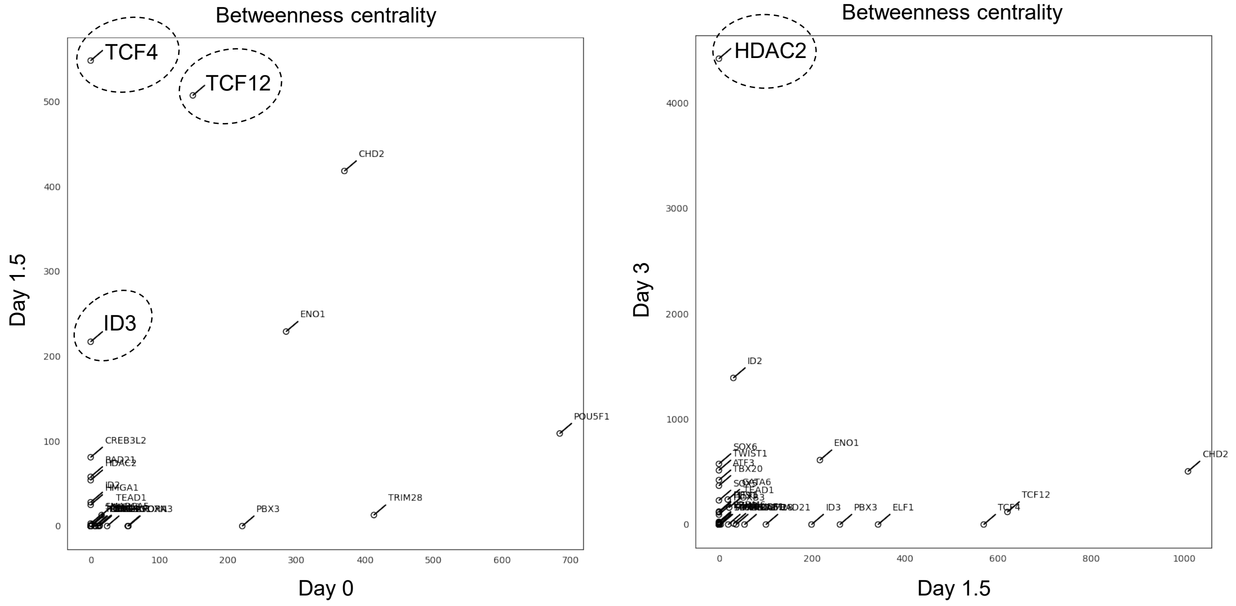

bioRxiv preprint doi: https://doi.org/10.1101/2025.06.06.657928; this version posted June 8, 2025. The copyright holder for this preprint (which was not certified by peer review) is the author/funder, who has granted bioRxiv a license to display the preprint in perpetuity. It is made available under aCC-BY 4.0 International license.

Figure S3 related to Figure 3. Identification of key regulators in RA-responsive mesodermal differentiation using CellOracle centrality analysis and in silico perturbation simulation. (A) CellOracle perturbation simulation showing the predicted effect of SP5 knockout on differentiation trajectories. Perturbation scores (PS) indicate where SP5 KO promotes (green, positive PS) or inhibits (magenta, negative PS) differentiation. (B) CellOracle network centrality analysis comparing TFs by betweenness centrality at Day 0 vs Day 1.5 cells (left) and Day 1.5 vs. Day 3 cells (right) of early CHIR cultures.

bioRxiv preprint doi: https://doi.org/10.1101/2025.06.06.657928; this version posted June 8, 2025. The copyright holder for this preprint (which was not certified by peer review) is the author/funder, who has granted bioRxiv a license to display the preprint in perpetuity. It is made available under aCC-BY 4.0 International license.

Fig. S4

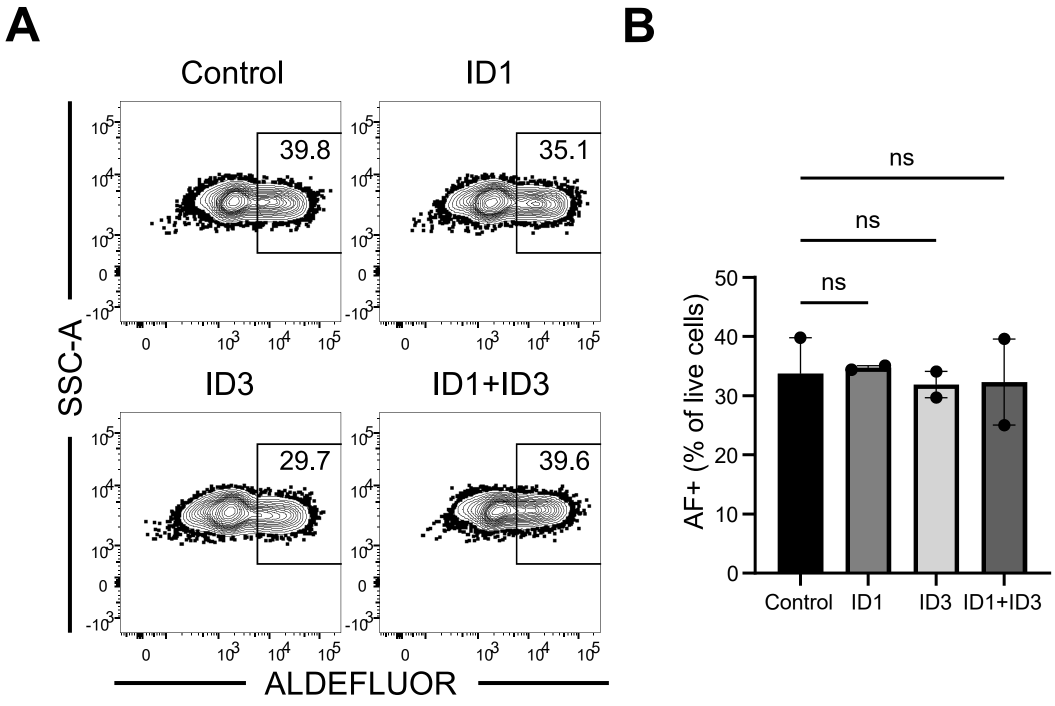

bioRxiv preprint doi: https://doi.org/10.1101/2025.06.06.657928; this version posted June 8, 2025. The copyright holder for this preprint (which was not certified by peer review) is the author/funder, who has granted bioRxiv a license to display the preprint in perpetuity. It is made available under aCC-BY 4.0 International license.

Figure S4 relate to Figure 5. Overexpression of ID proteins does not impair generation of AF+ cells. (A) Representative flow cytometry plots showing the differentiation of ALDEFLUOR+ progenitors on day 3 of differentiation. H1 cells were transduced with mCherry-expressing lentiviral vectors encoding ID1, ID3, and ID1 and ID3 in combination, or control (empty vector). Transduced cells were then subjected to the same differentiation protocol as above with CHIR 99021. (B) Quantification of ALDEFLUOR+ progenitor percentage. Statistical analyses were performed using paired 1-way ANOVA with Dunnett multiple comparisons test. Data are presented as mean ± SEM.

bioRxiv preprint doi: https://doi.org/10.1101/2025.06.06.657928; this version posted June 8, 2025. The copyright holder for this preprint (which was not certified by peer review) is the author/funder, who has granted bioRxiv a license to display the preprint in perpetuity. It is made available under aCC-BY 4.0 International license.

Table S1. Sequences of guide RNAs and PCR primers to generate amplicons for Nanopore sequencing used in Cas9-knockout validation experiments.

Name	Sequence	Source
B2M gRNA	GGCCACGGAGCGAGACAUCU	IDT
HDAC2 gRNA	GAUGUAUCAACCUAGUGCUG	IDT
TCF4 gRNA	CGAUGGAAAGUGGACAUCGG	IDT
TCF12 gRNA	AGUCGAUUAGGAGCCCAUGA	IDT
ID1 gRNA	CGGCAAGACAGCGAGCGGUG	IDT
ID3 gRNA	AUGUCGUCCAGCAAGCUCAG	IDT
B2M fwd seq	CGCTGGCTTGGAGACAGG	Sigma-Aldrich
B2M rev seq	GCAGCAGACAGGCTTACCCG	Sigma-Aldrich
HDAC2 fwd seq	GGTGCTGGAAAAGGCAAATACTATGCTG	Sigma-Aldrich
HDAC2 rev seq	CTGAACACATGAAGATACTGAGACACCAG	Sigma-Aldrich
TCF4 fwd seq	CCTCCACAGTTGATGCAAACCC	Sigma-Aldrich
TCF4 rev seq	GTAGCTCACTAGTCACTGATGGCTC	Sigma-Aldrich
TCF12 fwd seq	CCCTAGGGTTTTACAGACAGCCC	Sigma-Aldrich
TCF12 rev seq	CCTGTCCCTCCTGGACAAAG	Sigma-Aldrich
ID1 fwd seq	CGTATCTGCTTCGGGCTTCC	Sigma-Aldrich
ID1 rev seq	GCACGTAATTCCTCTTGCCCC	Sigma-Aldrich
ID3 fwd seq	GGCACCTCTGGACTCACTC	Sigma-Aldrich
ID3 rev seq	CGAGTCAGTGGCAAAAGCTCC	Sigma-Aldrich
